# Curcumin Nanocarriers in the Protection Against Iron- and Alcohol-Induced Oxidative Stress in a Cellular Model of Liver Disease

**DOI:** 10.3390/biology14050455

**Published:** 2025-04-23

**Authors:** Lucy Petagine, Mohammed G. Zariwala, Satyanarayana Somavarapu, Stefanie Ho Yi Chan, Vinood B. Patel

**Affiliations:** 1 Centre for Nutraceuticals, School of Life Sciences, University of Westminster, London W1W 6UW, UK; l.petagine@westminster.ac.uk (L.P.); m.zariwala@westminster.ac.uk (M.G.Z.); ho.chan.16@ucl.ac.uk (S.H.Y.C.); 2 Department of Pharmaceutics, UCL School of Pharmacy, London WC1N 1AX, UK; s.somavarapu@ucl.ac.uk

**Keywords:** alcohol, antioxidants, curcumin, iron, liver, mitochondria, oxidative stress, reactive oxygen species

## Abstract

During chronic alcohol misuse, hepatic iron overload occurs, leading to exacerbated oxidative stress and liver injury. The aim was to study formulations encapsulated with the antioxidant curcumin to assess their ability protect against oxidative stress in a model of alcohol-related liver disease (ALD) combined with iron. HepG2 (VL-17A) cells were treated with iron (50 µM) alone or with alcohol (200 to 350 mM) over 72 h and markers of oxidative damage, cell death, and mitochondrial function were assessed. Nanoformulations encapsulating curcumin were also studied. VL-17A cells treated with both ethanol and iron showed significant decreases in cell viability (64%, *p* < 0.0001) when compared to control, and a 56% decrease (*p* = 0.0279) when compared to iron-only treatment. Iron-alone treatment caused a 115% increase (*p* < 0.0001) in ROS at 48 h as well as increases of up to 118% when treated with 200 mM ethanol + 50 μM iron (*p* < 0.0001), compared to control DMEM. The study found that 10 µM curcumin DSPE-PEG increased cell viability by 17% and 41% when compared to control and iron treatment alone, respectively. Formulations reduced ROS by 36% (*p* = 0.0015) when compared to iron-alone treatment. In summary, encapsulated curcumin provided antioxidant capacity and reduced oxidative stress, demonstrating the therapeutic potential for curcumin formulations in ALD combined with iron dysregulation.

## Simple Summary

Excessive alcohol consumption can lead to liver damage, partly due to excess iron buildup in the liver. Excess iron in the liver can cause further damage due to oxidative stress, an imbalance of free radicals, and antioxidants in the body, which leads to cell damage. In this study, curcumin, a natural antioxidant found in turmeric, was developed into nanoformulations which enhance the properties of conventional drugs, to understand if curcumin can protect liver cells from damage. Liver cells were exposed to alcohol and iron to mimic the effects seen in alcohol-related liver disease. The results showed that alcohol and iron together significantly reduced cell survival and increased oxidative stress levels. However, when the curcumin nanoformulation was added, cell survival improved, and oxidative stress was reduced. Overall, curcumin nanoformulated drugs demonstrated strong protective effects against alcohol- and iron-induced liver cell damage. This suggests that curcumin formulations could be a promising future treatment option for liver disease associated with alcohol misuse.

## 1. Introduction

Alcohol-related liver disease (ALD) is a progressive disease, causing significant morbidity and mortality worldwide, and ranges from simple fatty liver to fibrosis and cirrhosis [1]. Although the stages of liver disease are well classified, the causes of alcohol-induced liver inflammation are complex. Multiple factors have been identified as causative factors to liver inflammation, resulting in oxidative damage and reactive oxygen species (ROS) production [2]. The liver plays a significant role in iron homeostasis as it stores iron as ferritin as well as in the labile iron pool (LIP) [3]. Alcohol consumption can lead to elevated liver iron levels [4]. Furthermore, during ALD, iron regulation may become disrupted, increasing the labile iron pool (LIP) in hepatocytes [5,6,7]. Alcohol-induced iron overload can cause further damage to the liver through free radical and inflammatory cytokine production, for example, NF-κB and TNF-α [8]. In animal models of ALD, Kupffer cells have been shown to contain elevated levels of iron which activate NF-κB (nuclear factor kappa B) and TNF-α (tumour necrosis factor alpha) [4,9,10]. Iron overload is common in fibrosis, cirrhosis, and end stage liver disease [11,12], and liver iron content has also been shown to be negatively correlated with ALD survival [13]. Therefore, due to increased oxidative stress occurring in ALD with concomitant iron dysregulation, the liver is more susceptible to oxidative damage and injury [13].

In general, therapeutic options for ALD remain limited and many clinical trials have been unsuccessful. Many limitations arise while using traditional drugs due to their low specificity, low bioavailability, and limited delivery to target sites. The use of nanocarriers may overcome the limitations of traditional drugs as they can enhance delivery to specific cell types via surface receptor binding. This specificity provided by nanocarriers can increase drug delivery and concentration at the target site as well as prevent metabolism and hydrolysis, leading to prolonged release [14,15]. Nanocarriers have previously been successfully researched in their antioxidant capacity, as well in their ability to significantly augment iron absorption, and are a potential delivery vehicle for nutritional applications [16].

Antioxidants can directly neutralise oxidative stress as well as scavenge free radicals and ROS, and therefore the use of antioxidants as well as methods to target the antioxidant system in the treatment of ALD have been considered. Curcumin, the main active compound in turmeric, has also been shown to have antioxidant properties via reduction in oxidative stress and ROS in animal models, as well as reduction in lipid accumulation in hepatocytes. Although natural compounds such as silymarin, quercetin, hesperidin, and berberine, when utilised in vitro and in vivo, may have shown some benefit for ALD, limitations still include low bioavailability, limited uptake, and low specificity [17]. Therefore, the aim of this study is to understand the potential of nanocarrier systems encapsulating antioxidants to protect against oxidative stress in a liver cell model of ALD combined with iron treatment.

## 2. Materials and Methods

### 2.1. Cell Culture

HepG2 (VL-17A) cells (kindly provided by Dr. Clemens, University of Nebraska, USA), which overexpress both ADH and CYP2E1 and have been previously characterised when studying fatty liver disease and alcohol toxicity [18,19,20,21], were cultured in Dulbecco’s modified Eagle medium (DMEM) (Lonza Ltd., Slough, UK) supplemented with 10% (*v*/*v*) foetal calf serum (FCS) (Gibco, London, UK) [21,22,23,24,25], 1% (*v*/*v*) (10 U/mL) penicillin/streptomycin, 1% (*v*/*v*) (2 mM) L-Glutamine and 50 μg/mL gentamycin sulphate (Lonza Ltd., Slough, UK). Cells were passaged using a 0.5× trypsin solution in phosphate-buffered saline (PBS) when they reached 70% confluency and viability of cells was assayed using trypan blue.

### 2.2. Cell Treatments

Cells were seeded according to the appropriate protocol and treated with alcohol (200 mM, 300 mM, and 350 mM) with or without iron (50 µM) or combinations of both in low-glucose (1.0 g/L) DMEM supplemented with 1% (*v*/*v*) FCS, 1% penicillin/streptomycin (100 U/mL), and 1% L-Glutamine (2 mM). The original methodology as stated by Clemens et al. was followed, where flasks were tightly sealed or cell culture plates were sealed to prevent ethanol evaporation [24,26]. Alcohol concentrations were chosen based on previous research whereby we showed that in HepG2 cells that overexpress ADH, 100 mM alcohol concentration led to a moderate decrease in cell viability and mitochondrial function [20]. Therefore, to induce further cellular injury reflecting stages of severe alcoholic hepatitis, higher concentrations of alcohol were characterised, up to 350 mM. Whilst these concentrations exceed clinical observations (up to 110 mM) the concentration corroborates other in vitro studies using HepG2 cell treatments at 171 mM [27], 184 mM [22], 300 mM [28], and 750 mM [29]; 100–800 mM in an L-02 hepatocyte line [30]; 200 mM in a mouse liver cell line [31]; and 300 mM in neuronal cells [32]. Cells were treated with iron (50 µM) with/without alcohol (200 to 350 mM). The varying treatments were studied using parameters of liver toxicity over 30 min to 72 h and assayed as below.

### 2.3. Cell Viability

Cells were seeded at 1 × 10^4^ cell/well and incubated overnight to allow adherence. Following treatment incubations with alcohol and/or iron, 5 mg/mL 3-(4,5-Dimethylthiazol-2-yl)-2,5-diphenyltetrazolium bromide (MTT) was added to each well and incubated at 37 °C for 2 h [21]. After incubation, the reagent was removed, and cells were incubated with 100 µL/well DMSO for 15 min at room temperature. The plates were then measured at 550 nm [20,21].

### 2.4. Reactive Oxygen Species Assay

The level of ROS production was assayed using a method adapted from Baldini et al. [33]. Cells were seeded at 1 × 10^4^ cells/well and incubated overnight to allow adherence. Following treatment incubations with alcohol and/or iron, cells were washed twice with PBS and incubated with 200 μL of 200 µM 2, 7-dichlorofluoresceindiacetate (DCF-DA) (Sigma-Aldrich, Gillingham, UK) at 37 °C for 30 min while protected from light. Following incubation, the substrate solution was removed, and cells were washed in PBS. After washing, 200 μL of fresh PBS was then added to each well and fluorescence was measured at excitation 485 nm and emission 535 nm [20,21].

### 2.5. Measurement of Apoptosis

To determine quantities of apoptotic cells after treatment, the Annexin VFITC/propidium iodide (PI) staining kit (BioLegend, London, UK) was used to stain cells with and measured by flow cytometry. Firstly, 3 × 10^5^ cells were seeded in 12-well plates and left overnight to adhere. Following adherence, cells were treated as described above with varying concentration of alcohol and/or iron over a 72 h period. After treatment, the supernatant of the cells was kept, and the cells were washed with PBS and detached from the plates with 200 µL trypsin (incubated at 37 °C for 5 min). To neutralise the action of trypsin, 800 µL of 10% FCS DMEM was then added, and the cell/supernatant mixture was then centrifuged at 400× *g* for 5 min. Cell pellets were resuspended in 500 µL of 1× Annexin V Binding Buffer and then stained with 5 µL of Annexin V-FITC and 5 µL PI, and analysed on the BD LSRFortessa™ (Wokingham, UK) (Ex = 488 nm, Em = 530 nm) using the FACSDiva software, Version 6.1.3 [20,21].

### 2.6. Measurement of Mitochondrial Respiration

HepG2 cells were plated in 24-well Seahorse MitoStress Assay cell plates at a density of 2 × 10^4^ cells per well. Following adherence, the cells were then treated with the previously described conditions of alcohol and/or iron. After the defined treatment lengths, the cells were washed with Seahorse Assay Medium (pH 7.4) containing 25 mM glucose and 1 mM sodium pyruvate. MitoStress drugs oligomycin (4 µM), FCCP (4 µM), and antimycin/Rotenone mixture (2.5 µM) were added, and the oxygen consumption rate (OCR) was measured using the Seahorse XFE Flux Analyzer (Agilent Technologies Ltd., Stockport, UK) under basal conditions. OCR values were normalised to total protein content, determined using the Bradford Assay, Bio-Rad protein assay kit (Bio-Rad Laboratories, Watford, UK), as per manufacturers conditions [20,21].

### 2.7. Preparation of Antioxidant Nanoformulations

The concentrations of curcumin were optimised based on previous studies from our research group [34,35] and existing literature [36,37]. In preclinical studies for assessing absorption efficiency and assessing the potential of bioenhancement strategies, such as nanoformulations and liposomal encapsulations, concentrations of 10 µM curcumin have been observed as optimal concentrations and therefore was chosen to ensure measurable and reproducible effects while minimising cytotoxicity.

All nanoformulations were prepared using a modified thin-film hydration method as previously described [38,39]. 1,2-Distearoyl-sn-glycero-3-phosphoethanolamine-Poly(ethylene glycol) (DSPE-PEG) was dissolved in 10 mL methanol, as well as 10 mg curcumin. Nanoformulations were formulated as 100% DSPE-PEG (blank formulations) and 10 mg curcumin with 90% DSPE-PEG. A rotary evaporator (Buchi Rotavapor^®^ R-100, Flawil, Switzerland) was then used to evaporate the methanol (200 rpm and 80 °C) under vacuum. Once a thin film was achieved, it was then hydrated with 10 mL of Milli-Q water and mixed at 80 °C then sonicated using a VWR Ultrasonic cleaner bath USC300T (VWR International Limited, Lutterworth, UK) for a further 5 min. The solution was then filtered through a sterile 0.45 μm filter to remove any unloaded curcumin [34,35,40,41].

### 2.8. Size and Surface Charge of Nanoformulations

The size and surface charge of nanoformulations were measured by dynamic light scattering (DLS) in terms of ZAve hydrodynamic diameter, polydispersity index (PDI) and zeta potential (§), using the Zetasizer Nano ZS (Malvern Instruments, Malvern, UK). In total, 1 mL of the nanoformulated sample was pipetted into the zeta potential DTS1070 folded capillary cell (Malvern Panalytical, Malvern, UK). Zeta potential was calculated via electrophoretic mobility using Malvern data analysis software Version 5.3 following the Helmholtz–Smoluchowski equation [34,35,40,41].

### 2.9. Determination of Drug Loading and Entrapment Efficiency

Drug loading and entrapment efficiency of nanoformulations were measured by UV–Visible spectrophotometer (Cary Series UV–Vis spectrophotometer, Agilent Technologies, Santa Clara, CA, USA) using free drug calibration curves. Curcumin was measured at 424 nm [34,35,40,41]. The percentage of drug loading and entrapment efficiency were calculated using the following equations:

Drug loading (%) = (total weight of entrapped drug)/(total weight of all raw materials) × 100

Encapsulation efficiency (%) = (determined mass of drug entrapped within nanocarriers)/(actual mass of drug) × 100

### 2.10. Statistical Analysis

Results were analysed using either one- or two-way ANOVA using GraphPad Prism Version 10.1.0 (Boston, MA, USA). Data were tested for normality using the Shapiro–Wilk test and confirmed for parametric analyses. Statistical analysis was followed by Tukey’s multiple comparisons post hoc test. Data are expressed as mean ± standard deviation (SD) and *p* values ≤ 0.05 were considered statistically significant.

## 3. Results

**Figure 1:**
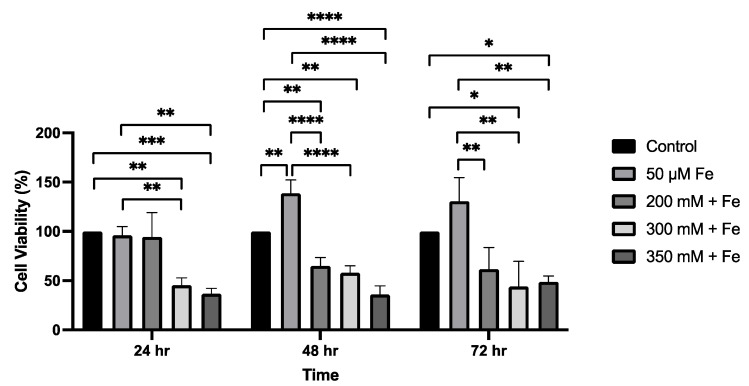
The effect of ethanol and iron exposure on cell viability over a 72 h period. Cells were seeded in 96-well plates and treated with 200 mM, 300 mM, and 350 mM ethanol as well as 50 μM iron. The viability of cells was determined by the MTT assay and measured at 24 h, 48 h, and 72 h. Data are presented as percentage from the control. Results are presented as mean ± SD (*n* = 3). * *p* ≤ 0.05, ** *p* ≤ 0.01, *** *p* ≤ 0.001, **** *p* ≤ 0.0001.

To model liver injury, VL-17A cells were treated with iron alone plus with ethanol for 24 to 72 h. At 24 h, ethanol and iron treatment in combination led to a 55% decrease (*p* = 0.0023) in cell viability at 300 mM ethanol + 50 μM iron, and a 63% decrease (*p* = 0.0008) when treated with 350 mM ethanol + 50 μM iron. At 24 h, treatment with ethanol and iron also produced significant differences when compared to the iron only treatment (Figure 1). At 48 h, ethanol and iron treatment combined led to a 35% decrease in cell viability at 200 mM ethanol + 50 μM iron (*p* = 0.0044), a 42% decrease (*p* = 0.0012) when treated with 300 mM ethanol + 50 μM iron, and a 64% decrease (*p* < 0.0001) when treated with 350 mM ethanol + 50 μM iron, when compared to the control (Figure 1). Similarly, treatment with ethanol and iron produced significant differences when compared to the iron-only treatment. At 72 h, ethanol and iron treatment produced significant changes when compared to the control and iron only. Treatment with 300 mM ethanol + 50 μM iron produced a 56% decrease (*p* = 0.0279) and 350 mM ethanol + 50 μM iron produced a 51% decrease (*p* = 0.0448), respectively, when compared to iron only (Figure 1).

**Figure 2:**
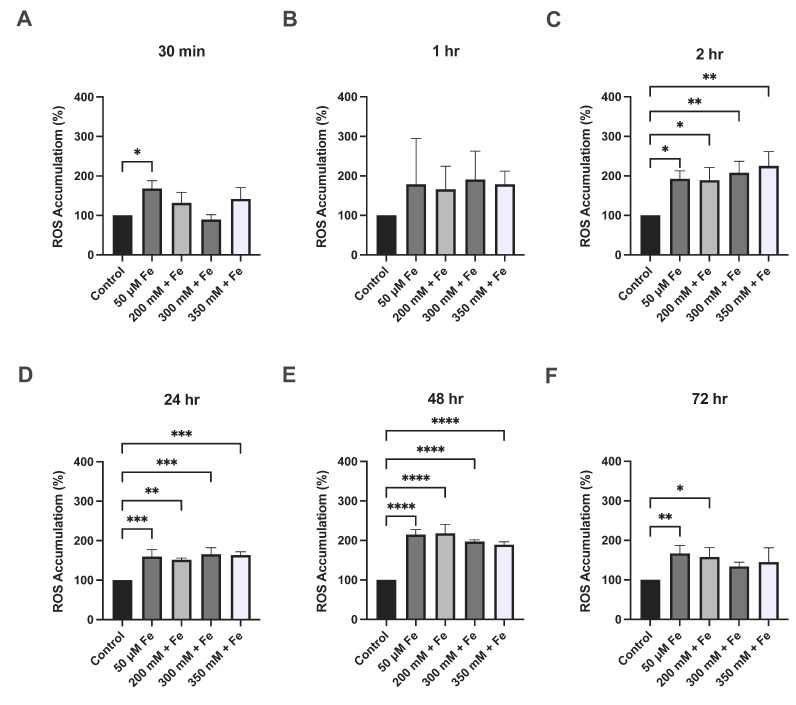
The effect of ethanol and iron exposure on ROS accumulation over a 72 h period. (**A**) The percentage of ROS accumulation at 30 min, (**B**) percentage of ROS accumulation at 1 h, (**C**) percentage of ROS accumulation at 2 h, (**D**) percentage of ROS accumulation at 24 h, (**E**) percentage of ROS accumulation at 48 h, and (**F**) percentage of ROS accumulation at 72 h. Cells were seeded in 96-well plates and treated with 200 mM, 300 mM, and 350 mM ethanol as well as 50 μM iron. ROS accumulation was determined by the DCFDA assay and measured using fluorescence at 30 min, 1 h, 2 h, 24 h, 48 h, and 72 h. Data are presented as percentage from the control. Results are presented as mean ± SD (*n* = 3). * *p* ≤ 0.05, ** *p* ≤ 0.01, *** *p* ≤ 0.001, **** *p* ≤ 0.0001.

Increased ROS production and oxidative stress is a common feature of ALD. After 30 min, iron treatment alone led to a significant increase (68%) in ROS (*p* = 0.0149); however, no changes were observed with ethanol and iron treatment combined. At 2 h, iron treatment alone led to a 92% increase (*p* = 0.0122) in ROS; 200 mM ethanol + 50 μM iron combined led to an 89% increase (*p* = 0.0154) in ROS; a 108% increase (*p* = 0.0043) was observed when cells were treated with 300 mM ethanol + 50 μM iron; and a 125% increase (*p* = 0.0014) when treated with 350 mM ethanol + 50 μM iron (Figure 2). Iron treatment only led to a 60% increase (*p* = 0.0007) at 24 h and a 115% increase at 48 h (*p* < 0.0001), whereas at 24 h, 200 mM ethanol + 50 μM iron led to a 52% increase (*p* = 0.0020) in ROS; a 66% increase (*p* = 0.0003) was observed with 300 mM ethanol + 50 μM iron treatment; and a 63% increase (*p* = 0.0004) was noted with 350 mM ethanol + 50 μM iron treatment, when compared to the control (Figure 2). At 48 h, a 118% increase (*p* < 0.0001) in ROS was observed when cells were treated with 200 mM ethanol + 50 μM iron; a 97% increase (*p* < 0.0001) when treated with 300 mM ethanol + 50 μM iron, and an 89% increase (*p* < 0.0001) when treated with 350 mM ethanol + 50 μM iron, when compared to the control (Figure 2). At 72 h, iron only increased ROS by 71% (*p* = 0.0044) and 200 mM ethanol + 50 μM iron caused a 69% increase (*p* = 0.0137) when compared to the control. No significant changes were observed when comparing ethanol and iron combination treatments to the iron-only treatment across all time points.

**Figure 3:**
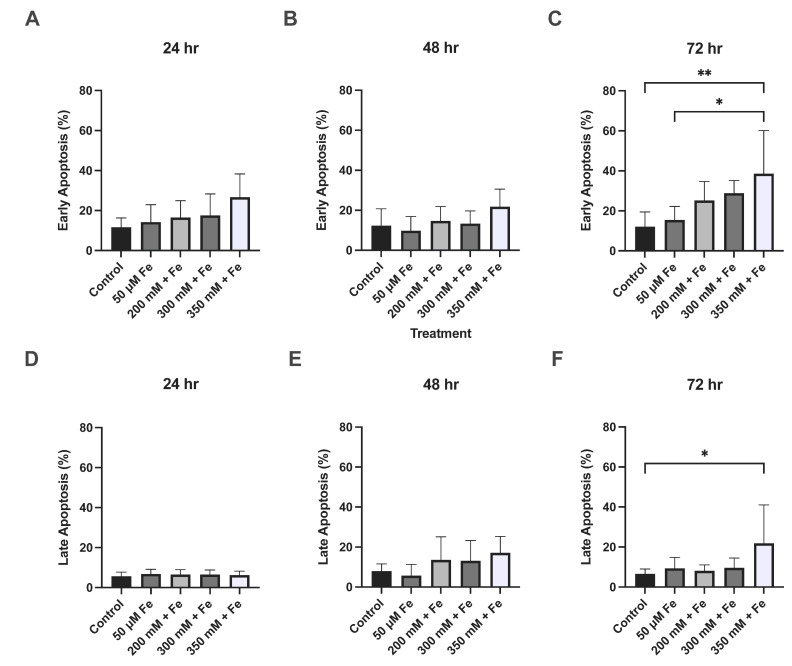
The effect of ethanol and iron exposure on apoptosis over a 72 h period. (**A**) The percentage of cells in early apoptosis at 24 h, (**B**) percentage of cells in early apoptosis at 48 h, (**C**) percentage of cells in early apoptosis at 72 h, (**D**) percentage of cells in late apoptosis at 24 h, (**E**) percentage of cells in late apoptosis at 48 h, and (**F**) percentage of cells in late apoptosis at 72 h. Cells were seeded in 12-well plates and treated with 200 mM, 300 mM, and 350 mM ethanol as well as 50 μM iron. Apoptosis was assessed at 24 h, 48 h, and 72 h using the Annexin VI kit and measured using flow cytometry. Data are presented as percentage of positive cells. Results presented as mean ± SD (*n* = 3). * *p* ≤ 0.05, ** *p* ≤ 0.01.

Significant changes were observed in both early and late apoptosis at 72 h. Treatment with 350 mM ethanol + 50 μM iron caused 39% of cells to undergo early apoptosis compared to 12% in the control group (*p* = 0.0057) and 15% in the iron treatment (*p* = 0.0267). Treatment with 350 mM ethanol + 50 μM also led to a total of 22% of cells in late apoptosis (*p* = 0.0478) (Figure 3).

**Figure 4:**
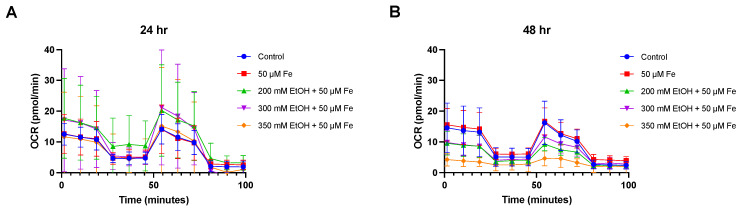
Mitochondrial oxygen consumption rate after ethanol and iron exposure at 24 h and 48 h. (**A**) Oxygen consumption rate at 24 h and (**B**) oxygen consumption rate at 48 h. Cells were seeded in 24-well plates and treated with 200 mM, 300 mM, and 350 mM ethanol as well as 50 μM iron. Oxygen consumption rate was assessed over 48 h using Seahorse XF24 analyser. Results are presented as mean of replicates ± SD (*n* = 3). OCR: oxygen consumption rate.

**Figure 5:**
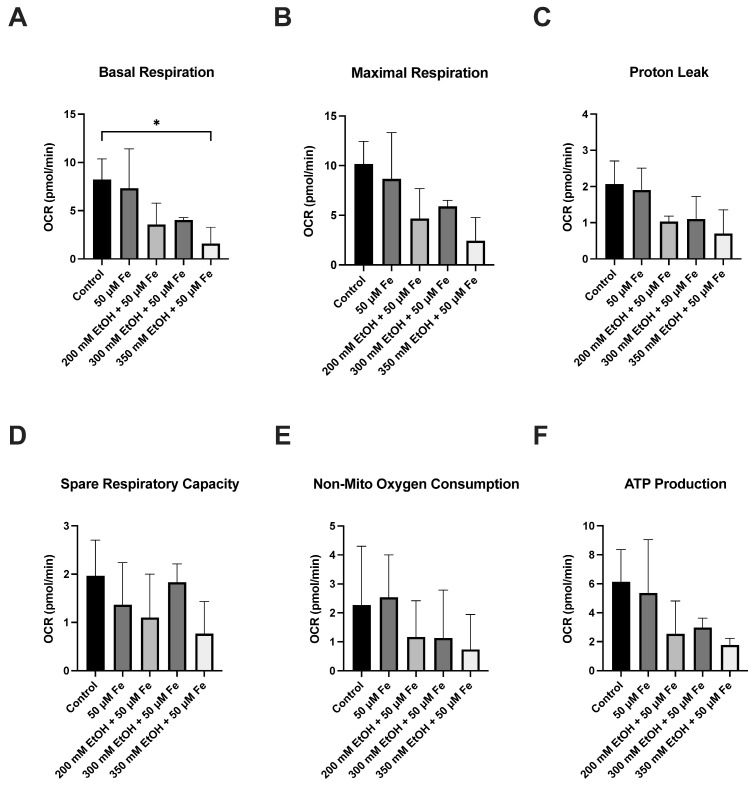
The effect of ethanol and iron on mitochondrial oxidative phosphorylation parameters at 24 h. (**A**) Basal respiration, (**B**) maximal respiration, (**C**) proton leakage, (**D**) spare respiratory capacity, (**E**) non-mitochondrial oxygen consumption, and (**F**) ATP production. Cells were seeded in 24-well plates and treated with 200 mM, 300 mM, and 350 mM with 50 μM iron. Oxygen consumption rate was assessed at 24 h using the Seahorse XF24 analyser. The results are presented as mean of replicates ± SD (*n* = 3). OCR: oxygen consumption rate. * *p* ≤ 0.05.

**Figure 6:**
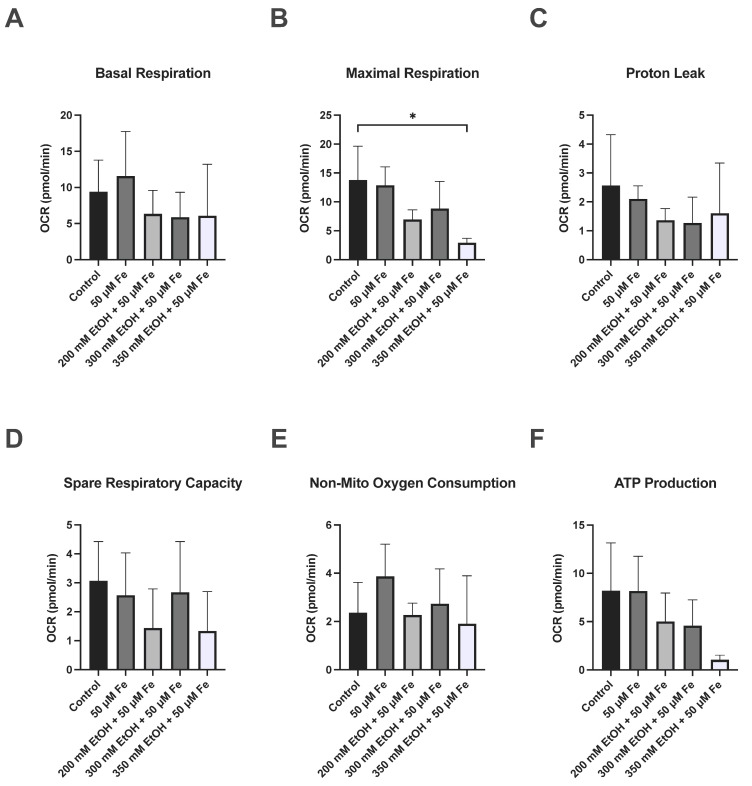
The effect of ethanol and iron on mitochondrial oxidative phosphorylation parameters at 48 h. (**A**) Basal respiration, (**B**) maximal respiration, (**C**) proton leakage, (**D**) spare respiratory capacity, (**E**) non-mitochondrial oxygen consumption, and (**F**) ATP production. Cells were seeded in 24-well plates and treated with 200 mM, 300 mM, and 350 mM with 50 μM iron. Oxygen consumption rate was assessed at 24 h using the Seahorse XF24 analyser. The results are presented as mean of replicates ± SD (*n* = 3). OCR: oxygen consumption rate. * *p* ≤ 0.05.

The respiratory function of mitochondria was evaluated by measuring the oxygen consumption rate of ethanol and iron treatment on VL-17A cells (Figure 4, Figure 5 and Figure 6). Significant decreases were observed in basal respiration at 24 h (*p* = 0.0433) and maximal respiration at 48 h (*p* = 0.0326) in the 350 mM ethanol + 50 μM iron-treated cells. No significant changes were observed in any other parameters.

Novel nanocarrier delivery systems were assessed for their protective properties against iron and ethanol cellular damage. The antioxidant curcumin was nanoformulated in carrier system DSPE-PEG and used to assess changes in cell viability and ROS production. Nanoformulations demonstrated high encapsulation efficiency (78.25%), as well as high drug loading of 7.11%. Curcumin DSPE-PEG nanocarriers had a mean diameter of 8.40 nm, as well as low polydispersity index values (<0.5) and low negative surface charges (−15.20) (Table 1).

**Figure 7:**
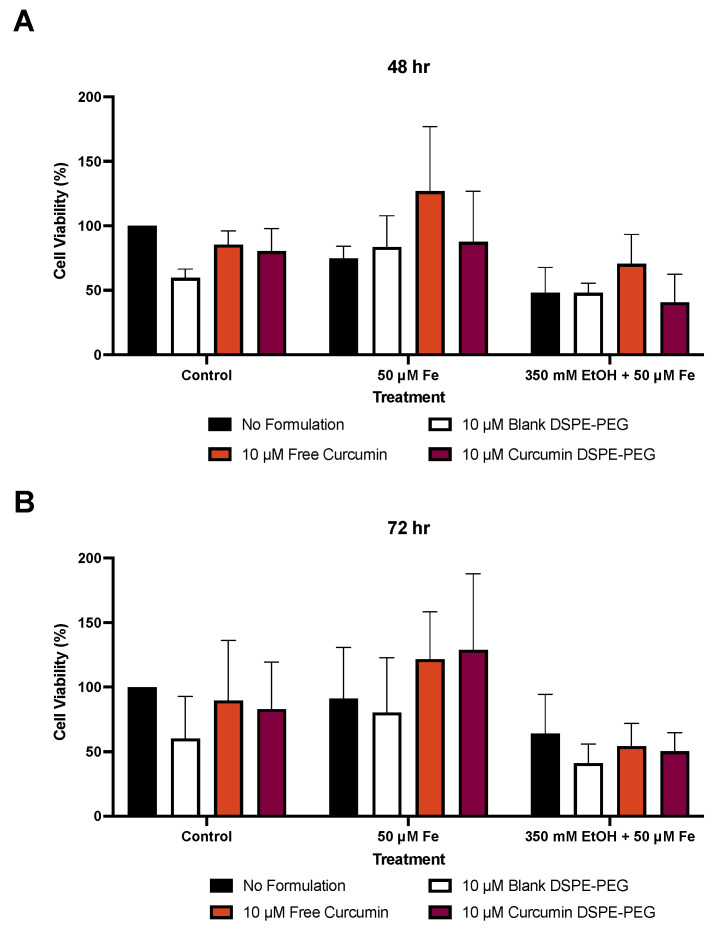
The effect of a 3 h pre-treatment of nanoformulated curcumin on ethanol- and iron-induced cell damage. (**A**) The percentage of cell viability at 48 h and (**B**) percentage of cell viability at 72 h. Cells were seeded in 96-well plates and co-treated with 350 mM ethanol and/or 50 μM iron as well as 3 h pre-treatment of formulations. The viability of cells was determined by an MTT assay and measured at 48 and 72 h. Data are presented as a percentage of the control. Results are presented as mean ± SD (*n* = 3).

At 48 h, 50 μM iron caused a 25% reduction in viability and 350 mM ethanol + 50 μM iron caused a 52% reduction in cell viability, when compared to the control DMEM. Blank formulations alone reduced viability by 40%, free drug (FD) curcumin by up to 15%, and nanoformulated curcumin by up to 20%. However, when treated with 10 μM curcumin DSPE-PEG + 50 μM iron, cell viability was increased by 17% in comparison to 50 μM iron only at 48 h. Also, at 72 h, 10 μM curcumin DSPE-PEG + 50 μM iron increased viability by 41% when compared to 50 μM iron treatment alone. Nanoformulations were unable to increase cell viability against 350 mM ethanol + 50 μM iron treatment at 72 h (Figure 7).

**Figure 8:**
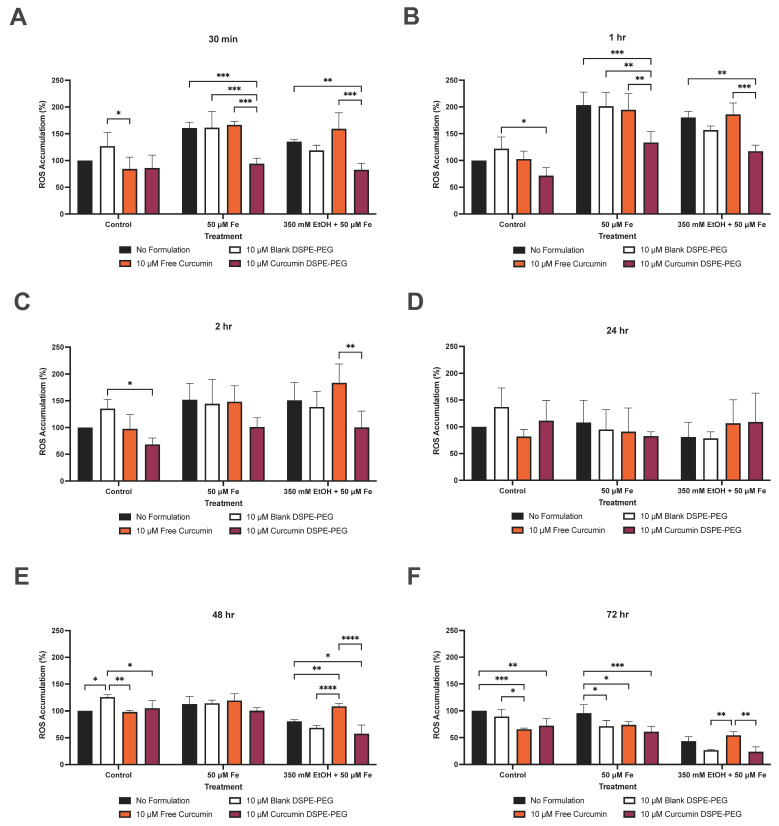
The effect of a 3 h pre-treatment of nanoformulated curcumin on ethanol- and iron-induced ROS production. (**A**) ROS production at 30 min, (**B**) ROS production at 1 h, (**C**) ROS production at 2 h, (**D**) ROS production at 24 h, (**E**) ROS production at 48 h, and (**F**) ROS production at 72 h. ROS production was determined by DCFDA assay. Cells were seeded in 96-well plates and co-treated with 350 mM ethanol and/or 50 μM iron as well as 3 h pre-treatment of formulations. Data are presented as a percentage of the control. Results are presented as mean + SD (*n* = 3) * *p* ≤ 0.05, ** *p* ≤ 0.01, *** *p* ≤ 0.001, **** *p* ≤ 0.0001.

Protection against oxidative stress was then assessed via ROS production over 72 h. At 30 min, pre-treatment with 10 μM curcumin DSPE-PEG reduced ROS by 41% (*p* = 0.0005) when compared to iron only treatment, and 39% (*p* = 0.0070) when compared to 350 mM ethanol + 50 μM iron treatment. At 1 h, pre-treatment with 10 μM curcumin DSPE-PEG reduced ROS by 34% (*p* = 0.0050) when compared to iron-only treatment, and 35% (*p* = 0.0122) when compared to 350 mM ethanol + 50 μM iron treatment. At 2 h, pre-treatment with 10 μM curcumin DSPE-PEG reduced ROS by 34% (*p* = 0.0050), when compared to iron-only treatment and by 35% (*p* = 0.0122), when compared to 350 mM ethanol + 50 μM iron treatment (Figure 8). At 48 h, decreases in ROS were also observed with pre-treatment with 10 μM curcumin DSPE-PEG. Moreover, 10 μM curcumin DSPE-PEG reduced ROS by 11% when compared to iron-only treatment, and by 29% (*p* = 0.0468) when compared to 350 mM ethanol + 50 μM iron treatment. At 72 h, pre-treatment with 10 μM curcumin DSPE-PEG reduced ROS by 36% (*p* = 0.0015) when compared to iron-only treatment, and by 45%, when compared to 350 mM ethanol + 50 μM iron treatment (Figure 8).

## 4. Discussion

The development of ALD is complex and is believed to involve multiple factors, including ROS, activation of inflammatory cells, gut dysbiosis, and iron overload. Despite some known mechanistic features of ALD, treatment options remain limited. In the current study, we were focussed on the combined effects of ethanol and iron, and whether antioxidant formulations could ameliorate oxidative damage. Ethanol and iron combined led to reductions in cell viability, while iron alone and combined with ethanol caused increases in ROS production, suggesting both alcohol and iron cause further detrimental effects on the liver. Iron overload is well documented in ALD patients, where iron is known to cause an increase in hydroxyl radical production, [42] and this study showed that iron caused further damage to the liver via the increase in ROS production possibly via the Fenton reaction. Recently, it has been demonstrated even mild alcohol consumption can elevate iron stores and >2 alcoholic drinks per day has been associated with an increased risk of iron overload [6]. There is significant evidence to suggest that both iron and alcohol alone can cause oxidative stress and lipid peroxidation and therefore, a combination of both alcohol and iron can exacerbate disease processes via an increased production of proinflammatory cytokines and free radicals. Increased iron in Kupffer cells in animal studies with experimentally induced ALD has also shown downstream activation of NF-κB, causing an increase in proinflammatory cytokines such as TNF-α [9,10]. This evidence, along with the toxic effects of iron demonstrated in this study of a reduction in cell viability and elevated ROS production, shows that iron has a pathogenic effect.

As oxidative stress is a major contributor to the pathogenesis of ALD, methods to enrich the antioxidant system have been considered for the treatment of this disease. Vitamins E/C, N-acetylcysteine (NAC), S-adenosyl methionine (SAMe), and betaine are antioxidants which moderate ROS. SAMe is a precursor of glutathione (GSH), the main cellular antioxidant [43,44]. Depleted SAMe and GSH levels have been documented in ALD, rendering antioxidant systems defective [45,46]. As antioxidants can regulate levels of catalase, superoxide dismutase, GSH, glutathione peroxidase, and glutathione reductase, their benefit in ALD therapeutics provides plausible reason. Curcumin has been shown to have numerous pharmacological benefits in vivo, including reductions in liver enzymes after curcumin therapy, and improved liver steatosis in patients with non-alcoholic fatty liver disease [47,48]. In clinical trials, curcumin formulations (500 mg/day equivalent to 70 mg curcumin) significantly reduced liver fat content (78.9%) compared to 27.5% improvement in the placebo group at 8 weeks, as well as reducing BMI, cholesterol, triglycerides, and liver enzymes [49]. In animal models of fatty liver, 60 mg/kg curcumin decreased malondialdehyde levels and increase glutathione peroxidase activity [50], whilst 50 mg/kg decreased ROS and increased superoxide dismutase and sirtuin-1 [51], suggesting curcumin can reduce oxidative stress and lipid peroxidation, via its antioxidant properties and protective effects against cellular damage. Therefore, antioxidant compounds such as curcumin were investigated in this study as they possess protective effects on chronic diseases [52,53,54,55], increase antioxidant enzyme capacity [56], and reduce inflammation and fibrosis [57,58,59].

Previous research has found beneficial effects of nanocarriers due to their ability to enhance stability, solubility, bioavailability, and delivery of the encapsulated compounds to specific cells [60]. The study aim was to investigate the protective effects of antioxidants encapsulated in DSPE-PEG nanocarrier systems against oxidative stress in ALD and iron treatment. Concentrations of nanocarriers were used at doses previously published in our group and by others [34,35,61]. Curcumin release from DSPE-PEG micelles is expected to follow diffusion-controlled kinetics, consistent with previous studies on hydrophobic drugs in similar formulations [62,63].

Whilst the free drug curcumin and curcumin DSPE-PEG were able to protect against iron-mediated loss of cell viability, this effect was not translated to the combined damaging effects of ethanol and iron treatment (Figure 7). The lack of prevention could be due to the toxicity of the ethanol metabolite, acetaldehyde. Alcohol toxicity initially arises due to oxidative stress, but the injury is then sustained due to elevated acetaldehyde levels, which can perturb mitochondrial function, leading to necroapoptotic cell death [64,65].

There are limited investigations of curcumin in alcohol toxicity. One study showed moderate reductions in hepatic inflammation in an in vivo model of chronic ethanol consumption with 20 mg/kg curcumin [66], whereas a single oral dose of ethanol 0.5 mL/kg body weight lowered acetaldehyde concentrations, not ethanol, following 30 mg administration of curcumin [67]. However, further investigations are required into whether curcumin can modulate acetaldehyde levels in this model system.

Curcumin possesses diketone and phenolic groups which allow it to scavenge free radicals [68]. In addition, curcumin is widely known for its iron-chelating properties, which have been implicated in its antioxidant effects. Previous studies have shown that curcumin can bind iron ions, reducing their pro-oxidant activity and suppressing ROS generation at concentrations up to 50 μM [69,70].

In the present study, 10 μM curcumin DSPE-PEG nanocarriers were able to reduce ROS production in both iron-only, and iron and ethanol treatments at earlier (30 min to 1 h) time points more significantly than later time points (72 h), compared to without curcumin (Figure 8), likely through the modulation of iron availability for the Fenton reaction; 10 μM curcumin DSPE-PEG also reduced ROS to a greater extent than curcumin FD, suggesting that encapsulation improves the efficacy of curcumin. This important finding confirms the ability of curcumin to reduce ROS. However, in our study, encapsulated curcumin did not significantly reduce iron-induced ROS levels at all time points or completely prevent ROS production. This discrepancy may arise from differences in curcumin bioavailability, as nanoformulations alter drug interaction with metal ions. Additionally, the curcumin concentration and iron overload conditions in our system may differ from prior studies, where higher concentrations were used. Further direct iron-binding assays are needed to confirm the extent of curcumin’s chelation in our DSPE-PEG formulation.

Treatment in animal models has shown that curcumin treatment led to a reduction in iron accumulation in both the liver and spleen, and also significantly reduced lipid peroxidation and nitric oxide levels [71]. Mechanisms contributing to the effect of curcumin in iron overload has been suggested to be due to the modulation of iron metabolism and iron-related proteins. Curcumin has been shown to repress the synthesis of hepcidin, an important regulator of iron homeostasis [72,73].

The specific mechanisms these antioxidants protect against oxidative stress is thought to be due to their ability to modulate of antioxidant signalling and mitochondrial function. Previous research suggests signalling pathways such as those in hepatic fibrosis (TGF-β1/Smad), apoptosis (JNK1/2-ROS), and inflammation (NF-κB) as well as antioxidant signalling can be altered by curcumin [74]. The pentose phosphate pathway may also be altered by curcumin. The pentose phosphate pathway enzymes produce NADPH, which is required for the conversion of oxidised glutathione to reduced GSH via glutathione reductase which enables cells to counterbalance oxidative stress [75]. In animal models, research has shown that long-term iron toxicity can strongly affect genes and enzymes associated with the pentose phosphate pathway [75].

Curcumin has many mechanisms of action including anti-inflammatory, antioxidant properties, and anti-apoptotic roles. Another key mechanism that curcumin can target is oxidative stress via reduced expression of monocyte chemoattractant protein 1 (MCP-1) as well as reduction in oxidative stress assessed by oxidative changes in hepatocyte DNA [76]. The hepatoprotective effects of curcumin have also been linked to its antioxidant properties and its activation of the nuclear factor erythroid 2-related factor 2 (Nrf2)/Kelch-MCP/antioxidant response element pathway, as well as its influence on inflammatory and apoptotic mediators [68]. Curcumin has been shown to modulate the Nfr2 pathway and mitogen-activated protein kinase pathways, which are involved in antioxidant defences, inflammation, and apoptosis [77].

### Considerations and Future Work

This study suggests that curcumin has beneficial effects on oxidative stress in the liver caused by both ethanol and iron injury in ALD. Whilst we also investigated the mechanistic effects of iron and high alcohol concentration leading to increased apoptosis and mitochondrial dysregulation, future studies need to assess whether curcumin is able to ameliorate these effects. DSPE-PEG nanocarriers were selected for their proven stability, enhanced curcumin solubility, and improved bioavailability. However, DSPE-PEG does not actively target the liver, relying instead on passive accumulation in hepatocytes and Kupffer cells via reticuloendothelial system uptake. Future work could explore liver-targeting modifications, such as glycyrrhizin-functionalised nanocarriers, which have demonstrated enhanced hepatic accumulation through asialoglycoprotein receptor recognition [78]. Whilst the study is a proof-of-concept investigation focussing on the most damaging concentrations of iron and alcohol, future studies will focus on a range of iron and ethanol concentrations, at various antioxidant nanoformulations (curcumin or n-acetylcysteine) [61] concentrations to determine the optimal protective dose–response effect. The capability of curcumin to chelate iron requires further investigation to understand the mechanisms in relation to mitochondrial function, autophagy and apoptosis in iron overload in ALD. Further work is also required understand the relationship between ROS and cell death, and to develop antioxidant formulations that can target acetaldehyde and apoptotic pathways.

## 5. Conclusions

In this study, VL-17A cells were characterised as a model to investigate the effects of alcohol exposure and iron treatment. Measurements of oxidative damage and mitochondrial function were assessed, and results show that iron treatment caused significant cell toxicity when combined with ethanol, with a dose-dependent reduction in cell viability observed over time, increases in ROS production and apoptosis as well as alterations to mitochondrial oxygen consumption. Antioxidant nanocarriers were shown to either enhance or match the protective effects of the free drugs investigated in this study in terms of ROS reduction. Overall, this study demonstrates that curcumin was successfully encapsulated into DSPE-PEG carriers and were observed to have a higher antioxidant capacity providing protection against oxidative stress in a cellular model of ALD and iron treatment. Whilst this study demonstrates the promising effects of nanoformulated antioxidants, these compounds require further biophysical and mechanistic investigations to assess their protective capacity in combined iron and alcohol treatment in liver disease.

## Figures and Tables

**Table 1:** Nanoformulation characteristics.

Active Ingredient (10 mg)	Polymer (100 mg)	Hydrodynamic Diameter (nm)	Polydispersity Index	Zeta Potential (mV)	Drug Loading (%)	Encapsulation Efficiency (%)
Curcumin	100% DSPE-PEG	8.40 (±1.160)	0.43 (±0.05)	−15.20 (±1.65)	7.11 (±0.00)	78.25 (±0.04)

Hydrodynamic diameter (d), polydispersity index (PDI), surface charge, drug loading (DL) and encapsulation efficiency (EE) of curcumin and ascorbyl palmitate (AP) DSPE-PEG nanoformulations prepared at 80 °C (data presented as mean ± SD, *n* = 3).

## Data Availability

The data presented in this study are available from the corresponding author upon reasonable request.

## References

[1] Mathurin P., Hadengue A., Bataller R., Addolorato G., Burra P., Burt A., Caballeria J., Cortez Pinto H., Day C.P., Forrest E.H. (2012). EASL Clinical Practical Guidelines: Management of Alcoholic Liver Disease. J. Hepatol..

[2] Cichoz-Lach H., Michalak A. (2014). Oxidative stress as a crucial factor in liver diseases. World J. Gastroenterol..

[3] Anderson G.J., Frazer D.M. (2005). Hepatic iron metabolism. Semin. Liver Dis..

[4] Harrison-Findik D.D. (2007). Role of alcohol in the regulation of iron metabolism. World J. Gastroenterol..

[5] Maras J.S., Maiwall R., Harsha H.C., Das S., Hussain M.S., Kumar C., Bihari C., Rastogi A., Kumar M., Trehanpati N. (2015). Dysregulated iron homeostasis is strongly associated with multiorgan failure and early mortality in acute-on-chronic liver failure. Hepatology.

[6] Ioannou G.N., Dominitz J.A., Weiss N.S., Heagerty P.J., Kowdley K.V. (2004). The effect of alcohol consumption on the prevalence of iron overload, iron deficiency, and iron deficiency anemia. Gastroenterology.

[7] Zanninelli G., Loréal O., Brissot P., Konijn A.M., Slotki I.N., Hider R.C., Cabantchik Z.I. (2002). The labile iron pool of hepatocytes in chronic and acute iron overload and chelator-induced iron deprivation. J. Hepatol..

[8] Ali N., Ferrao K., Mehta K.J. (2022). Liver Iron Loading in Alcohol-Associated Liver Disease. Am. J. Pathol..

[9] She H., Xiong S., Lin M., Zandi E., Giulivi C., Tsukamoto H. (2002). Iron activates NF-kappaB in Kupffer cells. Am. J. Physiol. Gastrointest. Liver Physiol..

[10] Xiong S., She H., Sung C.K., Tsukamoto H. (2003). Iron-dependent activation of NF-kappaB in Kupffer cells: A priming mechanism for alcoholic liver disease. Alcohol.

[11] Galaris D., Barbouti A., Pantopoulos K. (2019). Iron homeostasis and oxidative stress: An intimate relationship. Biochim. Biophys. Acta Mol. Cell Res..

[12] Mehta K.J., Farnaud S.J., Sharp P.A. (2019). Iron and liver fibrosis: Mechanistic and clinical aspects. World J. Gastroenterol..

[13] Ganne-Carrie N., Christidis C., Chastang C., Ziol M., Chapel F., Imbert-Bismut F., Trinchet J.-C., Guettier C., Beaugrand M. (2000). Liver iron is predictive of death in alcoholic cirrhosis: A multivariate study of 229 consecutive patients with alcoholic and/or hepatitis C virus cirrhosis: A prospective follow up study. Gut.

[14] Bartneck M., Warzecha K.T., Tacke F. (2014). Therapeutic targeting of liver inflammation and fibrosis by nanomedicine. Hepatobiliary Surg. Nutr..

[15] Bai X., Su G., Zhai S. (2020). Recent advances in nanomedicine for the diagnosis and therapy of liver fibrosis. Nanomaterials.

[16] Zariwala M.G., Farnaud S., Merchant Z., Somavarapu S., Renshaw D. (2014). Ascorbyl palmitate/DSPE-PEG nanocarriers for oral iron delivery: Preparation, characterisation and in vitro evaluation. Colloids Surf. B Biointerfaces.

[17] Yan J., Nie Y., Luo M., Chen Z., He B. (2021). Natural Compounds: A Potential Treatment for Alcoholic Liver Disease?. Front. Pharmacol..

[18] Lu Y., Cederbaum A.I. (2008). CYP2E1 and oxidative liver injury by alcohol. Free Radic. Biol. Med..

[19] Cederbaum A.I., Lu Y., Wu D. (2009). Role of oxidative stress in alcohol-induced liver injury. Arch. Toxicol..

[20] Gyamfi D., Everitt H.E., Tewfik I., Clemens D.L., Patel V.B. (2012). Hepatic mitochondrial dysfunction induced by fatty acids and ethanol. Free Radic. Biol. Med..

[21] Ghazali R., Mehta K.J., Bligh S.A., Tewfik I., Clemens D., Patel V.B. (2020). High omega arachidonic acid/docosahexaenoic acid ratio induces mitochondrial dysfunction and altered lipid metabolism in human hepatoma cells. World J. Hepatol..

[22] Wu H., Cai P., Clemens D.L., Jerrells T.R., Ansari G.A.S., Kaphalia B.S. (2006). Metabolic basis of ethanol-induced cytotoxicity in recombinant HepG2 cells: Role of nonoxidative metabolism. Toxicol. Appl. Pharmacol..

[23] Kumar S.M., Haridoss M., Swaminathan K., Gopal R.K., Clemens D., Dey A. (2017). The effects of changes in glutathione levels through exogenous agents on intracellular cysteine content and protein adduct formation in chronic alcohol-treated VL17A cells. Toxicol. Mech. Methods.

[24] Donohue T.M., Osna N.A., Clemens D.L. (2006). Recombinant Hep G2 cells that express alcohol dehydrogenase and cytochrome P450 2E1 as a model of ethanol-elicited cytotoxicity. Int. J. Biochem. Cell Biol..

[25] Valdés-Arzate A., Luna A., Bucio L., Licona C., Clemens D.L., Souza V., Hernandez E., Kershenobich D., Gutiérrez-Ruiz M.C., Gómez-Quiroz L.E. (2009). Hepatocyte growth factor protects hepatocytes against oxidative injury induced by ethanol metabolism. Free Radic. Biol. Med..

[26] Clemens D.L., Halgard C.M., Miles R.R., Sorrell M.F., Tuma D.J. (1995). Establishment of a recombinant hepatic cell line stably expressing alcohol dehydrogenase. Arch. Biochem. Biophys..

[27] Kim W., Jeong H.-S., Kim S.-C., Choi C.-H., Lee K.-H. (2021). Chronic Alcohol Exposure of Cells Using Controlled Alcohol-Releasing Capillaries. Cells.

[28] She X., Wang F., Ma J., Chen X., Ren D., Lu J. (2016). In vitro antioxidant and protective effects of corn peptides on ethanol-induced damage in HepG2 cells. Food Agric. Immunol..

[29] Qian Z.-J., Chen M.-F., Chen J., Zhang Y., Zhou C., Hong P., Yang P. (2021). Intracellular ethanol-mediated oxidation and apoptosis in HepG2/CYP2E1 cells impaired by two active peptides from seahorse (Hippocampus kuda bleeler) protein hydrolysates via the Nrf2/HO-1 and akt pathways. Food Sci. Nutr..

[30] Yang C.-F., Zhong Y.-J., Ma Z., Li L., Shi L., Chen L., Li C., Wu D., Chen Q., Li Y.-W. (2018). NOX4/ROS mediate ethanol-induced apoptosis via MAPK signal pathway in L-02 cells. Int. J. Mol. Med..

[31] Na A.-Y., Yang E.-J., Jeon J.M., Ki S.H., Song K.-S., Lee S. (2018). Protective Effect of Isoliquiritigenin against Ethanol-Induced Hepatic Steatosis by Regulating the SIRT1-AMPK Pathway. Toxicol. Res..

[32] Stoica S.I., Onose G., Pitica I.M., Neagu A.I., Ion G., Matei L., Dragu L.D., Radu L.-E., Chivu-Economescu M., Necula L.G. (2023). Molecular Aspects of Hypoxic Stress Effects in Chronic Ethanol Exposure of Neuronal Cells. Curr. Issues Mol. Biol..

[33] Baldini P., De Vito P., Vismara D., Bagni C., Zalfa F., Minieri M., Di Nardo P. (2005). Atrial Natriuretic Peptide Effects on Intracellular pH Changes and ROS Production in HEPG2 Cells: Role of p38 MAPK and Phospholipase D. Cell Physiol. Biochem..

[34] Mursaleen L., Chan S.H.Y., Noble B., Somavarapu S., Zariwala M.G. (2023). Curcumin and N-Acetylcysteine Nanocarriers Alone or Combined with Deferoxamine Target the Mitochondria and Protect against Neurotoxicity and Oxidative Stress in a Co-Culture Model of Parkinson’s Disease. Antioxidants.

[35] Mursaleen L., Somavarapu S., Zariwala M.G. (2020). Deferoxamine and Curcumin Loaded Nanocarriers Protect Against Rotenone-Induced Neurotoxicity. J. Park. Dis..

[36] Shahiduzzaman M., Dyachenko V., Khalafalla R.E., Desouky A.Y., Daugschies A. (2009). Effects of curcumin on Cryptosporidium parvum in vitro. Parasitol. Res..

[37] Premanand C., Rema M., Sameer M.Z., Sujatha M., Balasubramanyam M. (2006). Effect of curcumin on proliferation of human retinal endothelial cells under in vitro conditions. Investig. Ophthalmol. Vis. Sci..

[38] Tyrrell Z.L., Shen Y., Radosz M. (2010). Fabrication of micellar nanoparticles for drug delivery through the self-assembly of block copolymers. Prog. Polym. Sci..

[39] Somavarapu S., Sornsute A., Trill J., Abdelhakim H.E., Goonatilaka D., Pannala A.S., Taylor K.M. (2023). Engineering artesunate-loaded micelles using spray drying for pulmonary drug delivery. J. Drug Deliv. Sci. Technol..

[40] Chan S.H.Y., Sheikh K., Zariwala M.G., Somavarapu S. (2023). Dry powder formulation of azithromycin for COVID-19 therapeutics. J. Microencapsul..

[41] Schiano E., Novellino E., Fernández M.M.G., Lorinczova H.T., Tenore G.C., Iannuzzo F., Patel V.B., Somavarapu S., Zariwala M.G. (2023). Antioxidant and Antidiabetic Properties of a Thinned-Nectarine-Based Nanoformulation in a Pancreatic β-Cell Line. Antioxidants.

[42] Milic S., Mikolasevic I., Orlic L., Devcic E., Starcevic-Cizmarevic N., Stimac D., Kapovic M., Ristic S. (2016). The Role of Iron and Iron Overload in Chronic Liver Disease. Med. Sci. Monit..

[43] Cederbaum A.I. (2010). Hepatoprotective effects of S-adenosyl-L-methionine against alcohol- and cytochrome P450 2E1-induced liver injury. World J. Gastroenterol..

[44] Lu S.C., Mato J.M. (2012). S-adenosylmethionine in Liver Health, Injury, and Cancer. Physiol. Rev..

[45] Lee T.D., Sadda M.R., Mendler M.H., Bottiglieri T., Kanel G., Mato J.M., Lu S.C. (2004). Abnormal Hepatic Methionine and Glutathione Metabolism in Patients with Alcoholic Hepatitis. Alcohol. Clin. Exp. Res..

[46] Shen Y., Huang H., Wang Y., Yang R., Ke X. (2022). Antioxidant effects of Se-glutathione peroxidase in alcoholic liver disease. J. Trace Elem. Med. Biol..

[47] Ebrahimzadeh A., Ebrahimzadeh A., Fooladshekan S., Mohseni S., Mohtashamian A., Babajafari S., Sohrabi Z. (2025). Therapeutic effects of curcumin supplementation on liver enzymes of nonalcoholic fatty liver disease patients: A systematic review and meta-analysis of randomized clinical trials. Food Sci. Nutr..

[48] Ngu M.H., Norhayati M.N., Rosnani Z., Zulkifli M.M. (2022). Curcumin as adjuvant treatment in patients with non-alcoholic fatty liver (NAFLD) disease: A systematic review and meta-analysis. Complement. Ther. Med..

[49] Rahmani S., Asgary S., Askari G., Keshvari M., Hatamipour M., Feizi A., Sahebkar A. (2016). Treatment of Non-alcoholic Fatty Liver Disease with Curcumin: A Randomized Placebo-controlled Trial. Phytother. Res..

[50] Mahmoud A.A., Abdelrahman A., Abd el Aziz H.O. (2018). Protective effect of curcumin on the liver of high fat diet-fed rats. Gene Rep..

[51] Lee D.E., Lee S.J., Kim S.J., Lee H.-S., Kwon O.-S. (2019). Curcumin Ameliorates Nonalcoholic Fatty Liver Disease through Inhibition of O-GlcNAcylation. Nutrients.

[52] Kunnumakkara A.B., Bordoloi D., Padmavathi G., Monisha J., Roy N.K., Prasad S., Aggarwal B.B. (2017). Curcumin, the golden nutraceutical: Multitargeting for multiple chronic diseases. Br. J. Pharmacol..

[53] Pourbagher-Shahri A.M., Farkhondeh T., Ashrafizadeh M., Talebi M., Samargahndian S. (2021). Curcumin and cardiovascular diseases: Focus on cellular targets and cascades. Biomed. Pharmacother..

[54] Garodia P., Hegde M., Kunnumakkara A.B., Aggarwal B.B. (2023). Curcumin, Inflammation, and Neurological disorders: How are they linked?. Integr. Med. Res..

[55] Rahmani A.H., Alsahli M.A., Aly S.M., Khan M.A., Aldebasi Y.H. (2018). Role of Curcumin in Disease Prevention and Treatment. Adv. Biomed. Res..

[56] Machado I.F., Miranda R.G., Dorta D.J., Rolo A.P., Palmeira C.M. (2023). Targeting Oxidative Stress with Polyphenols to Fight Liver Diseases. Antioxidants.

[57] Federico A., Dallio M., Loguercio C. (2017). Silymarin/Silybin and Chronic Liver Disease: A Marriage of Many Years. Molecules.

[58] Ferenci P. (2016). Silymarin in the treatment of liver diseases: What is the clinical evidence?. Clin. Liver Dis..

[59] Lv D.-D., Wang Y.-J., Wang M.-L., Chen E.-Q., Tao Y.-C., Zhang D.-M., Tang H. (2021). Effect of silibinin capsules combined with lifestyle modification on hepatic steatosis in patients with chronic hepatitis, B. Sci. Rep..

[60] Jabczyk M., Nowak J., Hudzik B., Zubelewicz-Szkodzińska B. (2021). Curcumin in Metabolic Health and Disease. Nutrients.

[61] Mursaleen L., Noble B., Chan S.H.Y., Somavarapu S., Zariwala M.G. (2020). N-Acetylcysteine Nanocarriers Protect against Oxidative Stress in a Cellular Model of Parkinson’s Disease. Antioxidants.

[62] Remsberg C.M., Zhao Y., Takemoto J.K., Bertram R.M., Davies N.M., Forrest M.L. (2012). Pharmacokinetic Evaluation of a DSPE-PEG2000 Micellar Formulation of Ridaforolimus in Rat. Pharmaceutics.

[63] Haeri A., Sadeghian S., Rabbani S., Anvari M.S., Lavasanifar A., Amini M., Dadashzadeh S. (2013). Sirolimus-loaded stealth colloidal systems attenuate neointimal hyperplasia after balloon injury: A comparison of phospholipid micelles and liposomes. Int. J. Pharm..

[64] Farfán Labonne B.E., Gutiérrez M., Gómez-Quiroz L.E., Fainstein M.K., Bucio L., Souza V., Flores O., Ortíz V., Hernández E., Kershenobich D. (2009). Acetaldehyde-induced mitochondrial dysfunction sensitizes hepatocytes to oxidative damage. Cell Biol. Toxicol..

[65] Petagine L., Everitt H., Sherwood R., Gyamfi D., Patel V.B. (2022). Time-Dependent Alterations in Liver Oxidative Stress due to Ethanol and Acetaldehyde. J. Ren. Hepatic Disord..

[66] Singh A.K., Jiang Y., Gupta S., Younus M., Ramzan M. (2013). Anti-Inflammatory Potency of Nano-Formulated Puerarin and Curcumin in Rats Subjected to the Lipopolysaccharide-Induced Inflammation. J. Med. Food..

[67] Sasaki H., Sunagawa Y., Takahashi K., Imaizumi A., Fukuda H., Hashimoto T., Wada H., Katanasaka Y., Kakeya H., Fujita M. (2011). Innovative preparation of curcumin for improved oral bioavailability. Biol. Pharm. Bull..

[68] Yashmi F., Fakhri S., Varnamkhasti B.S., Amin M.N., Khirehgesh M.R., Mohammadi-Noori E., Hosseini M., Khan H. (2024). Defining the mechanisms behind the hepatoprotective properties of curcumin. Arch. Toxicol..

[69] Rainey N.E., Moustapha A., Saric A., Nicolas G., Sureau F., Petit P.X. (2019). Iron chelation by curcumin suppresses both curcumin-induced autophagy and cell death together with iron overload neoplastic transformation. Cell Death Discov..

[70] Messner D.J., Sivam G., Kowdley K.V. (2009). Curcumin reduces the toxic effects of iron loading in rat liver epithelial cells. Liver Int..

[71] Badria F.A., Ibrahim A.S., Badria A.F., Elmarakby A.A. (2015). Curcumin Attenuates Iron Accumulation and Oxidative Stress in the Liver and Spleen of Chronic Iron-Overloaded Rats. PLoS ONE.

[72] Mohammadi E., Tamaddoni A., Qujeq D., Nasseri E., Zayeri F., Zand H., Gholami M., Mir S.M. (2018). An investigation of the effects of curcumin on iron overload, hepcidin level, and liver function in β-thalassemia major patients: A double-blind randomized controlled clinical trial. Phytother. Res..

[73] Jiao Y., Wilkinson J., Di X., Wang W., Hatcher H., Kock N.D., D’Agostino R., Knovich M.A., Torti F.M., Torti S.V. (2009). Curcumin, a cancer chemopreventive and chemotherapeutic agent, is a biologically active iron chelator. Blood.

[74] Lukkunaprasit T., Tansawet A., Boonmanunt S., Sobhonslidsuk A., McKay G.J., Attia J., Thakkinstian A. (2023). An updated meta-analysis of effects of curcumin on metabolic dysfunction-associated fatty liver disease based on available evidence from Iran and Thailand. Sci. Rep..

[75] Budak H., Ceylan H., Kocpinar E.F., Gonul N., Erdogan O. (2014). Expression of glucose-6-phosphate dehydrogenase and 6-phosphogluconate dehydrogenase in oxidative stress induced by long-term iron toxicity in rat liver. J. Biochem. Mol. Toxicol..

[76] Guariglia M., Saba F., Rosso C., Bugianesi E. (2023). Molecular Mechanisms of Curcumin in the Pathogenesis of Metabolic Dysfunction Associated Steatotic Liver Disease. Nutrients.

[77] Sarmiento-Ortega V.E., Moroni-González D., Diaz A., Brambila E., Treviño S. (2025). Curcumin Treatment Ameliorates Hepatic Insulin Resistance Induced by Sub-chronic Oral Exposure to Cadmium LOAEL Dose via NF-κB and Nrf2 Pathways. Biol. Trace Elem. Res..

[78] Zhang Q., Polyakov N.E., Chistyachenko Y.S., Khvostov M.V., Frolova T.S., Tolstikova T.G., Dushkin A.V., Su W. (2018). Preparation of curcumin self-micelle solid dispersion with enhanced bioavailability and cytotoxic activity by mechanochemistry. Drug Deliv..

